# Clothing and Diet of Children

**Published:** 1881-02

**Authors:** 


### CLOTHING AND DIET OF CHILDREN.

IT is sad to think that thousands of beautiful little children are yearly sacrificed through errors of dress as well as errors of diet. Out of every one hundred children born with sound constitutions, ninety-nine should and would reach manhood and womanhood, with reasonable care and attention. We know of many large families, the youngest members of which have reached their majority, where no deaths have occurred. The family of the Queen of England is conspicuous in this respect. Then we have known families, naturally in the enjoyment of good health, that have been gradually but completely exterminated, before the prime of life, by diseases and accidents that were entirely preventable. Parents who attempt to raise children, without having some knowledge of the laws of health, and some idea of what is to be done in the accidents and emergencies liable to arise at any moment, are guilty of criminal neglect toward their own offspring. Tears and regrets are unavailing, and ignorance the most unconsoling of all excuses, for a bereavement that could have been easily avoided. Still, we will repeat what we have said substantially many times before, feeling almost certain that not one parent in a hundred who reads this article will think of it again, unless, perhaps, when the shadow of death has fallen on one of the dear little ones, and then it may be too late.

The healthy infant has a stronger hold on life than any other living creature. But he is the most helpless of all, and has not even the instinct of self-preservation, which is common to the lowest order of beings. He cannot live a day without the care and attention that is inspired by natural affection. If this care and attention are not constantly guided by intelligence, the life of the helpless little creature is in peril. The observance of one short, simple rule, is all sufficient to insure the health and life of any child.

### KEEP IT ALWAYS COMFORTABLE.

Any parent or nurse who will strictly observe this rule can be safely intrusted with the care of infants.

To insure a perfect circulation of the blood—which means perfect health—the child should be clothed entirely in flannel underclothing, woolen stockings, and thick, but loose and comfortable, shoes. When taken out its clothing should be sufficient to protect it from the cold, so that the blood will not be chilled, but not clothing enough to induce perspiration, which would be quite as hazardous as chilliness. Scanty clothing, low at the neck, bare legs and thin shoes, however fashionable, are invitations to disease and death. Every parent should have sense enough to judge correctly as to the amount of clothing that may be essential to comfort each day, in summer or winter.

It is difficult to say which are more destructive to health ; errors in dress, or errors in diet.

I have never believed that child unfortunate who had to be raised on the milk of cows or goats. Such children are almost always the healthiest and the brighest.

During the first ten days of a child's life, the milk might be diluted one-quarter, possibly one-third, with water ; but less and less water should be used until at the age of two or three months the child should have pure rich milk, and all that it cares to have. There is no danger of overfeeding, but thousands of infants have been starved in the midst of plenty.

It must be remembered that the child digests its food more rapidly than the adult, and that it will only cry for food when it needs it ; therefore give it food often, and pretty freely. Its instincts are more unerring than your judgment.

Until a child is ten years old, it should be fed all the plain, substantial food it wants, and ten times a day if it wishes it. Only when a child eats so often and so much as to neglect its regular meals, should any restrictions be placed upon him.

It is absurd to oppose exact rules to the instincts of hunger when the individual is in health. "No one knows where the shoe pinches so well as he who wears it." No one can judge of the appetite so well as he who feels the pangs of hunger. I am acquainted with a lady who lost her only child, a beautiful boy, two years old, under circumstances that embittered the remaining years of her life. The mother was trying to carry out an absurd and dangerous theory she had about feeding children. Her boy was from birth allowed to eat but three times a day. He became, of course, liable to attacks of illness peculiar to an impoverished condition of the blood. One evening, after a very light supper, the child was sent to bed. About ten o'clock it awoke and asked for a piece of bread, which was refused by the mother. It moaned and begged pitifully for food until about midnight, when both mother and child fell asleep. Soon after daylight the mother awoke and took her boy in her arms, but he was dead ; starved to death through the ignorance of a mother who would gladly have given her life could she have brought her child back to life.

No living beings are so tortured by thirst as little children. They should have all the cold, fresh water they will drink every hour, from the time they are a day old until they are old enough to make their wants known.

